# Responses of *Issatchenkia terricola* WJL-G4 upon Citric Acid Stress

**DOI:** 10.3390/molecules27092664

**Published:** 2022-04-21

**Authors:** Xinyi Liu, Ying Tang, Weiyu Ning, Yihong Bao, Ting Luo, Jinling Wang

**Affiliations:** 1 School of Forestry, Northeast Forestry University, No. 26, Hexing Street, Harbin 150040, China; lxy01211128@163.com (X.L.); nuyoah0820@163.com (Y.T.); wangjinling2018@163.com (Y.B.); 2 Heilongjiang Weiliang Biological Technology Co., Ltd., No. 45, Yanxing Street, Harbin 150001, China; ningweiyu2010@163.com; 3 Key Laboratory of Forest Food Resources Utilization of Heilongjiang Province, No. 26, Hexing Street, Harbin 150040, China; 4 State Key Laboratory of Food Science and Technology, Nanchang University, No. 999, Xuefu Street, Nanchang 330047, China

**Keywords:** *Issatchenkia terricola* WJL-G4, citric acid stress, membrane permeability, membrane fatty acids

## Abstract

This study aimed to elucidate the responses of a novel characterized *Issatchenkia terricola* WJL-G4 against citric acid stress by performing physiological analysis, morphology observation, and structural and membrane fatty acid composition analysis. The results showed that under citric acid stress, the cell vitality of *I. terricola* WJL-G4 was reduced. The cell morphology changed with the unclear, uncompleted and thinner cell wall, and degraded the cell structure. When the citric acid concentration was 20 g/L, *I. terricola* WJL-G4 could tolerate citric acid and maintain the cell structure by increasing the intracellular pH, superoxide dismutase activity, and contents of unsaturated fatty acids. As the citric acid concentration was ≥80 g/L, the stress has exceeded the cellular anti-stress ability, causing substantial cell damage. The cell membrane permeability, the content of membrane lipids, malondialdehyde and superoxide anion increased, but the intracellular pH and superoxide dismutase activities decreased, accompanying the increase of citric acid concentrations. The findings of this work provided a theoretical basis for the responsive mechanism of *I. terricola* WJL-G4 under high concentrations of citric acid, and can serve as a reference for biological acid reduction in fruit processing.

## 1. Introduction

Acid-tolerant yeast refers to a type of yeast that can grow or metabolize under the condition of pH ≤ 4, when the pH of the fermentation broth is controlled within the range of 3.5–4.5, bacteria and most other miscellaneous bacteria are inhibited, while the yeast, including non-*Saccharomyces cerevisiae* and *Saccharomyces cerevisiae*, can still function normally [1]. In recent years, the important role of non-*S. cerevisiae* wine yeasts in wine production has been re-evaluated since non-*S. cerevisiae* have desired enological characteristics that are absent in *S. cerevisiae*, such as producing high levels of aroma compounds, etc. [2]. However, in the fermentation process, strains are often subjected to a variety of environmental stresses, including acid stress, hypertonic stress, oxidative stress and ethanol stress, etc. Then, cell viability, adaptability and function were reduced [3]. Previous research has demonstrated that acid stress may cause membrane disruptive effects, which, in the presence of oxygen, leads to endogenous production of superoxide free radicals and the changes of antioxidant enzymes activity, such as reactive oxygen species (ROS), superoxide dismutase (SOD), and catalase (CAT) [4,5,6].

In order to adapt to various stress environments, yeast must make corresponding stress responses. Wang et al. [7] revealed that under salt stress, the shape of *Zygosaccharomyces rouxii* cells changed from round to elliptical and the cell walls were ruptured. They also reported changes in intracellular pH of *Z. rouxii* during salt stress, and the results suggested that the intracellular pH sharply decreased from 6.11 to 4.83 when cells encountered salt stress [8]. ROS accumulation and oxidative damage were observed in the enological strains of *S. cerevisiae* during fermentation in high-sugar-containing media, mimicking the composition of grape must [9].

As the first barrier between the external environment and the intracellular medium, the membrane regulated the movement of substances entering or exiting the cells and catalyzed exchange reactions and was the primary target for damage induced by environmental stress [10]. As a response to ethanol, osmotic, and thermal stress, several changes occur in the cell membrane, particularly in the lipids and fatty acids (FA) of the membrane, to reduce the damage to cells [11,12]. Rodriguez-Vargas et al. [1] reported that cell membrane fluidity of *S. cerevisiae* decreased and its fatty acid composition changed in response to freezing and salt stress. Plasma membrane permeability was found to increase when *S. cerevisiae* cells were exposed to acetic acid stress and to decrease following adaptation to stress and growth resumption in acetic acid presence [13].

Any microorganism exposed to citric acid may induce a stress response. Lawrence et al. [14] found that citric acid inhibited growth of *S. cerevisiae*, and exposure to citric acid up-regulated the expression of many stress response genes and proteins, such as Bmh1p, Ssa1p, and Eno2p. Nielsen et al. [15] reported that the growth-inhibitory effect of citric acid on both *S. cerevisiae* and *Z. bailii* was increased when adjusting the pH from 3.0 to 4.5, and citric acid induced drastic changes in the primary energy metabolism of *S. cerevisiae* (i.e., it lowered the ethanol production and increased the glycerol production, and thus it reduced the ATP production). Likewise, Omori et al. [16] reported that the citric acid caused an increased glycerol production of *S. cerevisia*. However, the effects of citric acid on growth and metabolism of other yeast species remain widely unknown.

In our previous study, a novel characterized *I. terricola* WJL-G4 (Chinese patent No. 2019113316700) was isolated from red raspberry fruits, which exhibited a potent capability of degrading citric acid about 20 or 60 g/L in raspberry or lemon juice, and 20–200 g/L in citric acid medium [17,18], indicating a wide range of application prospects not only in fruit processing but also in the treatment of increasingly serious global ecological environment degradation, such as soil acidification and wastewater acidification [19,20]. However, the acid-tolerant responses and mechanism of this strain is still unclear.

To expand the application of *I. terricola* WJL-G4 in biological acid-lowering in fruit processing, ecological governance, and environmental protection, it is of great significance to clarify the response, self-defense ability of *I. terricola* WJL-G4 under citric acid stress. Therefore, in this study *I. terricola* WJL-G4 was cultured under high citric acid concentrations, aiming to investigate the changes of cellular physiological characteristics, membrane permeability, membrane fatty acids compositions and antioxidant enzyme activities. The results could provide theoretical basis for the acid-tolerant mechanism of yeast, enrich the research work of non-*S. cerevisiae* for bio-deacidification, and offer new strain for the development of fruit processing, industrial utility and ecological governance.

## 2. Results

### 2.1. Effect of Citric Acid on Cell Structure by TEM and SEM Observation

**Figure 1:**
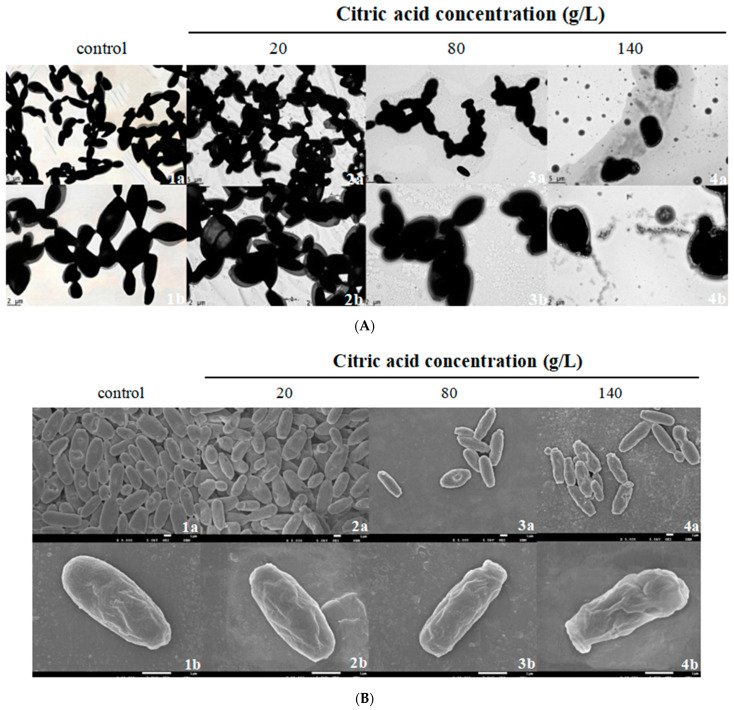
TEM and SEM images of extracellular morphology of *I. terricola* WJL G4 under different citric acid concentrations. Letters correspond to: (**A**): TEM images; (**B**): SEM images; 1, 2, 3, and 4: control, 20, 80, and 140 g/L citric acid; a: ×5000; b: ×20,000.

Cell integrity was critical in maintaining cell viability and metabolic function, particularly under stress [21]. Under the optimum pH values, citric acid declined cell viabilities of *I. terricola* WJL-G4 under high concentration (Supplementary Figure S1). To investigate the effect of citric acid on the cell integrity of *I. terricola* WJL-G4, the cell surface morphology and structure were analyzed by TEM observation. As shown in Figure 1A, in the absence of citric acid (control group), the cells were elliptical shape, with integral cell morphology of smooth surface and intact and clear cell walls. The cell morphology and structure of 20 g/L citric acid was similar to that of the control group. However, as the concentrations of citric acid increased, cellular structure began to degrade. The cells transformed into an irregular shape, while the cell walls became relatively thinner, and no longer intact and clear. In addition, oily liquid was observed under thinner cell wall when the citric acid content increased to 140 g/L. Similarly, Guan et al. [22] investigated the effects of osmotic stress on the morphology of *Pichia pastoris* by TEM, and found that cell wall of the *Ppyps7△* mutant *P. pastoris* became thinner and coarser. Wu et al. reported that after 1 h of lactic acid shock, the cell surface of the wildtype *Lactobacillus casei* became rough [23].

The change in cellular structure can be considered as a mean of adaptation of the cells to unfavorable environmental conditions. The differences in surface properties of cells subjected to different treatments (control, 20, 80 and 140 g/L citric acid) were investigated by SEM observation (Figure 1B). The cells in control group were elliptical shape with smooth surface and free of impurities. Compared to the control group, the cells cultured in citric acid content of 20 g/L showed slight folds and holes, while apparent folds and holes on the cell surface, and seriously deformed and rough surface were observed when concentrations of citric acid were 80 and 140 g/L. Moreover, with an increase of citric acid concentrations, the length of the short axis of the cells decreased and the morphology changed obviously. Liu et al. [24] reported that high-glucose stress induced apparent folds and holes on the outer membrane of *Zygosaccharomyces mellis* 6-7431. Wu et al. [25] found that acid or alkaline stresses induced the alterations in surface structures of *Salmonella enterica* serovar Typhimurium and changed cell surface from smooth appearance into slightly rough surface. The silimar changes were observed with *S. cerevisiae* in other study [26].

These results showed that an increase in citric acid concentrations likely affected *I. terricola* WJL-G4 cells, including their cell surface characteristics, cell integrity, cell shape, cell wall, and cell membrane.

### 2.2. Effect of Citric Acid on Intracellular pH

**Figure 2:**
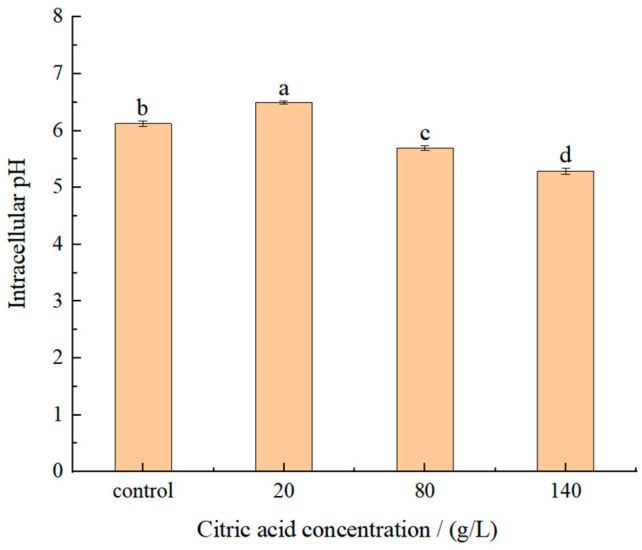
Intracellular pH in different citric acid concentrations of *I. terricola* WJL-G4. Error bars: SD (*n* = 3). Statistically significant differences (*p* < 0.05) were determined by one-way ANOVA with Duncan’s test and were indicated with different letters.

A fluorescent dye, 2′,7′-bis-(2-carboxyethyl)-5-(and-6)-carboxyfluorescein, acetoxymethyl ester (BCECF AM), could penetrate cell membranes. BCECF AM has no fluorescence and can be cleaved by intracellular esterase to form BCECF after entering the cell, thus being retained in the cell. BCECF can be excited to form green fluorescence under the condition of appropriate pH value, and can be used for the detection of intracellular pH level in animal tissues, plant cells, bacteria and yeast [27]. The Intracellular pH was determined from the ratio of the pH sensitive wavelength (490 nm) and the pH insensitive wavelength (440 nm). The results (Figure 2) showed that the intracellular pH decreased gradually with an increase of citric acid concentrations. Compared with control group the intracellular pH of *I. terricola* WJL-G4 was significantly higher when the concentration of citric acid was 20 g/L, indicating that yeasts could resist external stress by increasing the intracellular pH [28]. However, when the concentrations of citric acid were ≥80 g/L, the intracellular pH of strain was significantly lower than that of 20 g/L and control group. It indicated that under low concentration of citric acid stress, yeasts could resist external stress by increasing the intracellular pH [28]. When citric acid concentrations were ≥80 g/L, physiological regulation cannot be effectively carried out.

### 2.3. Effect of Citric Acid on Cell Membrane Permeability

**Figure 3:**
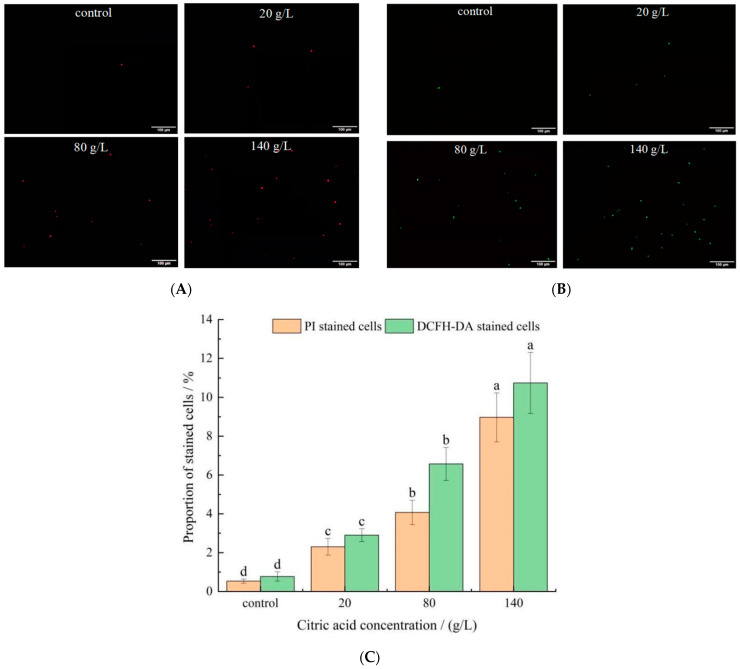
PI and DCFH-DA staining observation results. Error bars: SD (*n* = 3). Statistically significant differences (*p* < 0.05) were determined by one-way ANOVA with Duncan’s test and were indicated with different letters within each indicator. Letters correspond to the following: (**A**) fluorescence microscope observation pictures after PI staining. (**B**) Fluorescence microscope observation pictures after DCFH-DA staining. (**C**) The proportions of PI/DCFH-DA-stained cells in different citric acid concentrations of *I. terricola* WJL-G4.

Propidium iodide (PI) cannot pass through an intact cell membrane, but can stain nucleic acids through a damaged cell membrane. Therefore, the changes in cell membrane permeability can be indirectly reflected by comparing the changes in proportion of stained cells before and after cell staining. Representative images were presented in Figure 3A after staining with PI, and the changes of proportion of stained cells were shown in Figure 3C. Compared with control group (0.53 ± 0.11%), proportion of PI-stained cells in 20, 80, and 140 g/L citric acid treatments were increased to 2.30 ± 0.43, 4.07 ± 0.63 and 8.97 ± 1.26%, respectively. High proportion of non-permeabilized yeasts was detected in the control group. Conversely, with the concentrations of citric acid increased, the cell membrane permeability was enhanced, indicating that high concentrations of citric acid caused damage to the cell membrane, and increase apoptotic cells [29]. Câmara et al. [30] studied the oxidative stress on three dehydrated non-*Saccharomyces* yeast strains and found that the proportion of stained cells and the number of apoptosis cells increased significantly after dehydration.

It is well known that one of the reasons for cell death during stress is partially due to oxidative stress and accumulation of ROS inside cells [31]. The oxidant probe, 2′,7′-dichloroflfluorescein diacetate (DCFH-DA), was used to determine the ROS accumulation inside yeast cells. Representative images were presented in Figure 3B after staining with DCFH-DA, and the changes of proportion of stained cells were shown in Figure 3C. The proportion of DCFH-DA stained cells in the control group, 20, 80, and 140 g/L citric acid treatments were 0.77 ± 0.24, 2.90 ± 0.33, 6.57 ± 0.85 and 10.74 ± 1.57%, respectively. *I. terricola* WJL-G4 cultivated in control group showed slight stain for fresh cells (Figure 3B,C), compared to that cultivated in citric acid medium. It suggested that cells accumulated significant ROS with increased citric acid concentrations. It was previously revealed that after ethanol treated, high proportion of ROS accumulation was also observed in budding yeast *S. cerevisiae* cells [32]. Moreover, enhanced metabolite flux through the photorespiratory pathway, caused by various abiotic stresses, leads to the over production of ROS in plants [33]. Tetrabromobisphenol A could induce cells oxidative stress of *S. cerevisiae* through the accumulation of intracellular ROS [34]. Dysregulated intracellular redox balance would lead to lipid peroxidation, the disorder of substance or energy metabolism [35].

The results above indicated that 20 g/L citric acid concentration has already produced acid-stress on *I. terricola* WJL-G4, although the cell morphology has not changed significantly.

### 2.4. Effect of Citric Acid on Membrane Fatty Acids Composition by GC-MS Analysis

Fourteen fatty acids were presented in the membrane of *I. terricola* WJL-G4 cells in control group. After cultivated in different citric acid concentrations, the composition of membrane fatty acids changed into twelve kinds (Table 1).

In control group, four kinds of saturated fatty acids (SFAs), including myristic acid, pentadecanoic acid, palmitic acid and stearic acid, were detected; among them, palmitic acid and stearic acid were the major SFAs. In addition, the major unsaturated fatty acids (USFAs) were (Z)-9-octadecenoic acid, linoleic acid and 11-octadecenoic acid. Under citric acid stress treatment, two kinds of SFAs (palmitic acid and stearic acid) and ten kinds of USFAs were detected. Among them, palmitic acid was the major SFA, while (Z)-9-octadecenoic acid and linoleic acid were the major USFAs.

Compared to the control group, myristic acid, pentadecanoic acid, and (Z)-hexadec-9-enoate acid were not detected, while (Z)-11-eicosenoic acid, 5,9-octadecadienoate acid and 6,10-octadecadienoate acid were detected as new components of fatty acids under citric acid stress. Moreover, the contents of (Z)-9-octadecenoic acid and linoleic acid were significantly increased, the contents of (9E)-hexadec-9-enoate acid and palmitic acid increased, while the contents of 11-octadecenoic acid and 10-hydroxy-octadecanoic acid decreased, compared to the control group. In general, with an increase of citric acid concentrations, membrane lipids contents increased significantly, mainly due to the contents increase of USFAs. The results suggested that citric acid changed the composition of membrane fatty acids.

### 2.5. Effect of Citric Acid on Antioxidative Enzyme System

**Figure 4:**
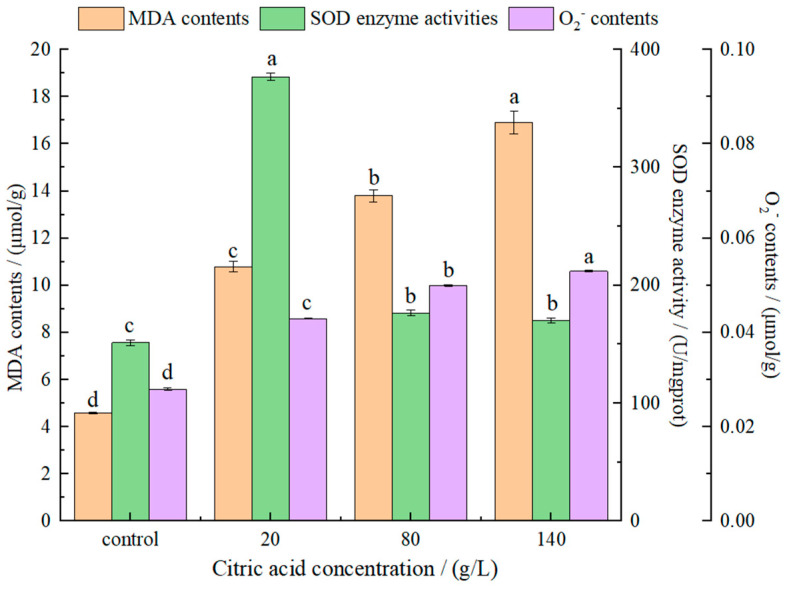
The effect of different citric acid concentrations on MDA contents, SOD activities and O_2_^−^ contents of *I. terricola* WJL-G4. Error bars: SD (*n* = 3). Statistically significant differences (*p* < 0.05) were determined by one-way ANOVA with Duncan’s test and were indicated with different letters within each indicator.

As one of the main products of cell lipid oxidation reaction, malonic dialdehyde (MDA) can be used as an important indicator of the strength of oxidation. Secondly, MDA can also react with some components in the cell to amplify the effect of ROS, thereby reducing membrane fluidity, which will eventually cause membrane integrity and structural damage [36]. MDA detection reflecting lipid peroxidation processes was shown in Figure 4. With an increase of citric acid concentrations, MDA contents increased gradually, indicating that the high concentrations of citric acid caused damage to the cell membrane. It also indicated that MDA contents in yeast was positively correlated with citric acid concentrations. Ma et al. [37] proved that MDA contents in *S. cerevisiae* cells was significantly enhanced under ethanol stress, resulting in increased lipid peroxidation or decreased antioxidant defense mechanism. Chi et al. [38] reported that the heat or oxidative stress resulted in a marked increase in MDA contents in *Pichia kudriavzevii* cells.

Exposure of *I. terricola* WJL-G4 cells to citric acid stress led to a significant increase in SOD activities (Figure 4), especially when the concentration of citric acid was 20 g/L. When citric acid concentrations were 80 g/L and 140 g/L, there were no significant difference in SOD between them, but were significantly lower than that of 20 g/L. The superoxide anion radical is the first radical formed by oxygen in the organism accepting an electron. It can be regarded as base in an aqueous solution and accept H^+^ to form protonated superoxide anion radical HOO· [39]. With an increase in citric acid concentrations, superoxide anion (O_2_^−^) contents increased gradually (Figure 4).

The results showed that the presence of citric acid increased the MDA contents, SOD activity and O_2_^−^ contents of *I. terricola* WJL-G4 compared with the control group.

## 3. Discussion

### 3.1. Intracellular pH as Stress Homeostasis Indicator for Yeast

The stability of intracellular pH plays an important role in the normal metabolism of microorganisms [40]. Studies have shown that acidic environment could reduce intracellular pH and increase intracellular ROS, thus reducing the survival rate of yeast cells under acidic environment [41].

In this study, the intracellular pH values showed a significant downward trajectory with an increase of citric acid concentrations, and the *I. terricola* WJL-G4 would have a stress response to increase intracellular pH when exposed to the citric acid concentration of 20 g/L. Nevertheless, when the concentrations of citric acid were ≥80 g/L, the intracellular pH values of strain decreased significantly. In conclusion, when the concentration of citric acid was 20 g/L, *I. terricola* WJL-G4 withstood the stress through physiological regulation by increasing the intracellular pH, which might be due to the increase in H^+^-ATPase activity [42].

Studies have shown that the intracellular pH value of cells was related to cell growth, cell adhesion, and cell endocytosis, the decreased of intracellular pH value was accompanied by the weakening of cell growth activity [43]. Under caprylic acid or acetic acid stress, the activity of *S. cerevisiae* intracellular H^+^-ATPase was enhanced to expel the excess H^+^ out of the cell, thus maintaining the stability of intracellular pH and reducing the damage to cell membrane [42]. The increase of intracellular H^+^-ATPase activity in *S. cerevisiae* under environmental stress was related to the expression of plasma membrane H^+^-ATPase encoding genes *PMA1* and *PMA2*, which could maintain the physiological homeostasis of the strain [1]. Besides, Wu et al. [44] found that the acid-stress evolved strains had higher acid tolerance when studying the acid resistance of *L. casei*, compared with the original strains, and that the intracellular pH of the evolved strains increased to maintain cell viability.

### 3.2. Impact of USFAs Levels on Stress Tolerance of Yeast

Fatty acids are the major constituents of membrane glycerolipids, and the distribution of USFAs and SFAs affects the membrane fluidity. The regulation of cell membrane fatty acid profiles is one of the effective ways for cells to combat environmental stress [10]. Under high pressure, most cells maintain their biochemical function by altering the membrane components [45]. Based on the present findings, changes in membrane fatty acid composition of *I. terricola* WJL-G4 might be caused by citric acid stress. However, the results obtained in this study clearly identify an increase in USFAs level as a response to exposure to high citric acid concentrations.

Similar results were reported in a study on *Debaryomyces hansenii* under salt stress, which showed that high NaCl concentrations resulted in an increase in the degree of unsaturation magnitude of fatty acids within the membrane, at 0.5 M NaCl, which is close to the salt concentration in sea water, a decrease in membrane fluidity was observed which correlated with the changes in lipid composition [46]. Nasution et al. reported that an increase in USFAs contents could increase the osmotic stress tolerance of *S. cerevisiae.* This was caused by expression of *OLE1*, which encoding delta-9 desaturase to generate unsaturated fatty acids [47]. It has been shown that heat stress tolerance of *S. cerevisiae* cells was enhanced by an increase in unsaturation of membrane fatty acids [48]. Also, the increase in oleic acid (C18:1) proportion observed in *S. cerevisiae* cell cultures containing ethanol was accompanied by a decrease in palmitoleic acid (C16:1) proportion [49], which suggested that ethanol raised the proportion of the USFAs [50]. Zheng et al. showed that the higher the contents of USFAs, the better the tolerance of *S. cerevisiae* to external stress, which was realized through the overexpression of gene *ELO1* [51].

On the other hand, Lindberg et al. found [52] that the saturated glycerophospholipids and sphingolipids appear to be key lipid classes in response to acetic acid stress. Under acetic acid stress, *Z. bailii* exhibited a considerable increase in the saturation of glycerophospholipids, as well as an increase in sphingolipids, and *Z. bailii* has highly adaptable to acetic acid exposure. However, our results seem to be inconsistent with this mechanism. Therefore, future work is warranted to address the interaction between fatty acid composition in menbrane and citric acid tolerance.

### 3.3. Yeast Stress Response Is Affected by SOD Levels

In our study, ROS, MDA, and O_2_^−^ were significantly accumulated under citric acid stress. SOD, a kind of antioxidant enzyme widely existing in animals, plants and microorganisms, has the effect of scavenging O_2_^−^ free radical and eliminate the toxicity of ROS to organisms, and plays an important role in protecting cells [53].

When the concentration of citric acid was 20 g/L, the SOD activity was increased obviously (2.49-fold compared to that of control, Figure 4), and when the concentrations of citric acid were 80 g/L and 140 g/L, SOD activities increased by 1.17-fold and 1.13-fold compared to that of control, respectively, indicating that the strain changed correspondingly in combat and tolerant the citric acid stress.

The increased SOD activities have been shown to protect *Arbuscular mycorrhizal* fungi from salinity injury and to improve their salt tolerance [54]. Studies on the effects of sodium chloride and cadmium on growth, oxidative stress and antioxidant enzyme activities of *Z. rouxii* showed that SOD activity increased and then decreased with an increase of NaCl or Cd^2+^ concentrations. Increased SOD activities may contribute to the high salt tolerance of *Z. rouxii*, but under harsh environmental conditions, Li et al. [55] observed that the SOD activities of *Z. rouxii* decreased, mainly due to the fact that the contents of O_2_^−^ in the system exceeded the elimination capacity, which was in agreement with our results. Lee et al. [56] studied the stress response mechanism of *Schizosaccharces pombe* under oxidative stress, and the results showed the expressing of a large number of genes encoding SOD were up-regulated, such as *sod1*^+^, and increased SOD activities, thereby avoiding damage to cells by oxidative stress. Moreover, Abrat et al. [42] studied the effect of acetic acid on survival and activity of SOD in different *S. cerevisiae* strains, and found that incubation of the yeast with 150–200 mM acetic acid caused the increase of SOD activities in the wild strain.

## 4. Materials and Methods

### 4.1. Yeast Cultivation

The yeast used in this study was screened from fresh red raspberry [17], named as *I. terricola* WJL-G4 (Chinese patent No. 2019113316700) after morphological and molecular biological identification, and stored at China General Microbiological Culture Collection Center (CGMCC), No. 18712.

*I. terricola* WJL-G4 was inoculated into ready-made basic liquid medium (1% citric acid, 0.01% magnesium sulfate and 0.5% yeast extract) in 100 mL/250 mL conical flasks, and then incubated as seed solution at 28 °C for 24 h with a shaking speed of 120 r/min. The seed solution was adjusted to a density of approximately 1 × 10^8^ CFU/mL and inoculated with an inoculum size of 1% (*v*/*v*) into 250 mL flask with 40 mL of citric acid medium (20, 80, and 140 g/L, 0.01% magnesium sulfate, 0.5% yeast extract, pH 3.0) and cultured at 28 °C, 120 r/min. Yeast extract magnesium sulfate dextrose (0.5% yeast extract, 0.01% magnesium sulfate, 2% dextrose, pH 3.0) was set as the control.

Cells were harvested after 15 h cultivation in different citric acid concentrations and control group medium, then centrifuged at 6000× *g* for 10 min, and washed twice serially with sterile water for subsequent analyses.

### 4.2. Transmission Electron Microscopy (TEM) Observation

The samples for TEM observation were prepared according to the methods described by Wang et al. [7] with slight modifications. Briefly, cells subjected to different treatments (control, 20, 80 and 140 g/L citric acid) were collected and fixed in 2.5% glutaraldehyde at room temperature for 3 h, washed and fixed in 1% osmium tetroxide for 1 h. Subsequently, the cells were washed, then followed by dehydration in a graded series of ethyl alcohol and twice washes in propylene oxide, dehydrated in acetone and examined with a transmission electron microscope (JEM-2100, JEOL, Tokyo, Japan).

### 4.3. Scanning Electron Microscope (SEM) Observation

SEM was measured as described by Zeng et al. [26] with some modifications. Briefly, cells fixed with 2.5% glutaraldehyde were washed twice with phosphate buffer (0.05 M, pH 7.0), and then dehydrated with 30, 50, 70, 90 or 100% concentrations of ethanol for 15 min to achieve absolute dry samples, and followed by specimen critical point drying. After coating with gold–palladium for 2 min, cells were evaluated by scanning electron microscope (JSM-7500F, JEOL, Tokyo, Japan).

### 4.4. Measurement of Intracellular pH

The intracellular pH values were determined and calibrated using the modified method reported by Breeuwer et al. [57]. The cells were washed twice with 50 mmol/L 4-(2-Hydroxyethyl)piperazine-1-ethanesulfonic acid potassium salt (HEPES-K) buffer (pH 8.0) and resuspended in an equal volume of HEPES-K buffer. Then added an aliquot (1 μL) of BCECF AM, and in a water bath at 30 °C for 20 min, then washed twice with 50 mmol/L phosphate buffer saline (PBS) buffer, and then resuspended in an equal volume of PBS buffer. Cells containing fluorescent probe were diluted to a concentration of approximately 1 × 10^8^ cells per mL in a 3 mL glass cuvette and placed in the stirred and thermostated cuvette holder of the spectrofluorometer. Fluorescence intensities were measured at excitation wavelengths of 490 and 440 nm by rapidly alternating the monochromator between both wavelengths. The emission wavelength was 525 nm, and the excitation and emission slit widths were 5 and 10 nm, respectively. The 490 to 440 nm ratios were corrected for back-ground signal due to buffer. The incubation temperature was 25 °C (unless indicated otherwise). At the end of each assay, the extracellular fluorescence signal (background) was determined by filtration of the cell suspension through a 0.22 μm membrane filter and measurement of the filtrate. The degree of fluorescence intensity was calculated as follows:

$$I=\frac{{\left(I_{490}\right)}_{total}-{\left(I_{490}\right)}_{filtrate}}{{\left(I_{440}\right)}_{total}-{\left(I_{440}\right)}_{filtrate}}$$

Note: (*I_490_*)*_total_*: Fluorescence intensity of yeast suspension at 490 nm.

(*I_490_*)*_filtrate_*: Fluorescence intensity of supernatant at 490 nm.

(*I_440_*)*_total_*: Fluorescence intensity of yeast suspension at 440 nm.

(*I_440_*)*_filtrate_*: Fluorescence intensity of supernatant at 440 nm.

*I*: Total fluorescence intensity.

Calibration curve was determined in buffers with pH values ranging from 4 to 7.2. The pH_in_ and pH_out_ were equilibrated by addition of nigericin (1 mM). Calibration curve was constructed by plotting the ratio of fluorescence intensities (emission wavelength 525 nm) at the excitation wavelengths of 490 and 440 nm as a function of pH. Intracellular pH was calculated from this calibration curve.

### 4.5. Measurement of Cell Membrane Permeability

Cell membrane permeability measurements were assessed using a fluorescence microscope after stained by PI and DCFH-DA.

#### 4.5.1. PI Fluorescent Staining Method

Cells were washed twice with 0.01 mol/L PBS buffer (pH 7.4). The number of cells in the suspension was adjusted to 4.55 × 10^8^ CFU/mL, then 0.5 mL PBS buffer was added into 0.2 mL cells suspension and mixed, and centrifuged (6000× *g* for 10 min). The pelleted cells that formed were then prefixed in 1.0% paraformaldehyde at 4 °C for subsequent analyses. After prefixing, cells were collected by centrifugation (6000× *g* for 10 min), then 0.5 mL PBS buffer was added and mixed, and centrifuged again. After keeping in a dark place for 15 min, 10 μL of PI (1 mg/mL) was added to the tube immediately, blended, and incubated at 37 °C for 15 min, then washed three times with 0.01 mol/L PBS buffer, resuspended with 200 μL of PBS, and performed fluorescence microscope observation [58].

#### 4.5.2. DCFH-DA Fluorescent Staining Method

Cells were washed twice with 0.01 mol/L PBS buffer (pH 7.4). The number of cells in the suspension was adjusted to 4.55 × 10^8^ CFU/mL, then 0.5 mL PBS buffer was added into 0.8 mL cells suspension and mixed, and centrifuged (6000× *g* for 10 min). After discarding the supernatant, 200 μL 10 μmol/L of DCFH-DA was added to the tube immediately and incubated at 37 °C for 20 min, then washed three times with 0.01 mol/L PBS buffer, resuspended with 200 μL of PBS. Observation with fluorescence microscope (CFM-880E, Pudan Optical Instrument Co., Ltd., Shanghai, China) [59].

### 4.6. Membrane Fatty Acids Analysis

Membrane lipids were extracted from 1 mL aliquots with 3.75 mL of chloroform-methanol (1:2, *v*/*v*) according to the modified method of Bligh and Dyer [60]. For fatty acid analysis, methyl esters were generated by acid-catalyzed esterification (2.5% [*v*/*v*] H_2_SO_4_ in dry methanol) at 70 °C for 2 h. Fatty acid methyl esters (FAMEs) were extracted with redistilled petroleum spirit and subsequently analyzed by gas chromatography-mass spectrometer (GC-MS) [61].

Gas chromatographic analysis was performed according to Zhang et al. [62]. One microliter of the concentrated FAME extract was assayed by a gas chromatograph interfaced with a mass spectrometer detector (7890A-7000B, Agilent Technologies Inc., Santa Clara, CA, USA) using capillary column Rxi-5 ms (30 m × 0.25 mm). The carrier gas was helium at a flow rate of 29.6 mL/min, the column pressure was 63.4 kPa, and the column flow was 0.5 mL/min. The temperatures used were 260 °C for the injection port and 280 °C for the detector. The temperature program was 100 °C isothermally for 1 min followed by 4 °C/min to 250 °C and then 250 °C isothermally for 5 min. FAMEs were identified by their mass spectra compared against a spectrum database. A known quantity of nonadecanoic acid methyl ester [C19:0] was used as an internal injection standard. The data shown represented means at least two independent samples, each analyzed in duplicate. The results were expressed as milligram lipid per gram dry biomass (mg/g) for each FA [63].

### 4.7. Antioxidant System Analysis

Crude enzyme solution in cells was extracted by the method of Saharan et al. [64]. The SOD activity and contents of MDA were measured by using assay kits (SOD test, and MDA test, Nanjing Jiancheng Bioengineering Institute, Jiangsu, China) [65]. Superoxide anion (O_2_^−^) accumulation inside yeast cells was determined according to the modified method reported by Ke et al. [66]. Briefly, hydroxylamine hydrochloride (0.1 mL, 10 mM) was added to 0.5 mL of 65 mM potassium phosphate buffer solution, then 100 μL of the extracted crude enzyme solution was added in, followed by water bath at 25 °C for 20 min, after that, 1 mL of 58 mM p-amino-phenylsulfonic acid and 1 mL of 7 mM α-naphthylamine were added and followed by water bath at 25 °C for 20 min, the absorbance of the sample was determined at 530 nm. Sodium nitrite was used as standard solution to calculate the contents of O_2_^−^.

### 4.8. Statistical Analysis

Each experiment was repeated three times unless otherwise stated, and the results were expressed as the means ± standard deviation. SPSS 20.0 (V.26.0, SPSS Inc., Chicago, IL, USA) was used to analyze variance, and Duncan’s multiple comparison test was used to analyze the differences between the experimental groups, *p* < 0.05 was considered statistically significant.

## 5. Conclusions

In conclusion, physiological approaches were used in the present study to investigate the responses of *I. terricola* WJL-G4 to citric acid stress to reveal its protective mechanisms. The results indicated that *I. terricola* WJL-G4 could tolerate and combat citric acid stress of 20 g/L through increasing intracellular pH, SOD activities, and contents of USFAs to maintain the cell viability. However, when the concentrations of citric acid were ≥80 g/L, the cell morphology changed significantly, the cell structure began to degenerate, the cell membrane permeability became larger, the contents of MDA and superoxide anion increased, but the intracellular pH and SOD activities decreased. This indicated that when the concentrations of citric acid were ≥80 g/L, the stress caused substantial damage to cells. Our results may facilitate the development of new strategies aimed at enhancing the industrial utility, ecological governance and environmental protection of *I. terricola* WJL-G4, at the same time, it provides theoretical guidance and research ideas for applications of non-*S. cerevisiae*. Additional studies should be undertaken to define the molecular mechanisms adaptation of *I. terricola* WJL-G4 under stressful conditions.

## Figures and Tables

**Table 1:** Types and contents of fatty acids in cell membranes of *I. terricola* WJL-G4.

Fatty Acid Types	Contents of Cell Membrane/(mg/g DW)			
	Control	20 g/L	80 g/L	140 g/L
Myristic acid	0.09 ± 0.01	-	-	-
Pentadecanoic acid	0.10 ± 0.02	-	-	-
(Z)-hexadec-9-enoate acid	0.43 ± 0.06	-	-	-
(9E)-hexadec-9-enoate acid	1.01 ± 0.42 ^b^	1.41 ± 0.17 ^b^	3.67 ± 0.63 ^a^	3.45 ± 0.11 ^a^
Palmitic acid	14.28 ± 1.68 ^c^	14.81 ± 0.34 ^bc^	18.72 ± 0.99 ^ab^	22.26 ± 0.08 ^a^
7,12-Octadecadienoate acid	0.19 ± 0.02	-	-	-
Linoleic acid	9.08 ± 1.09 ^b^	19.29 ± 0.46 ^a^	20.28 ± 1.73 ^a^	18.46 ± 0.72 ^a^
(Z)-9-Octadecenoic acid	16.61 ± 0.54 ^d^	32.10 ± 1.56 ^c^	49.53 ± 0.63 ^b^	58.17 ± 0.60 ^a^
11-Octadecenoic acid	8.00 ± 1.19 ^a^	3.86 ± 0.16 ^b^	3.24 ± 0.54 ^b^	2.64 ± 0.05 ^b^
Stearic acid	1.51 ± 0.09 ^a^	2.03 ± 0.08 ^a^	1.98 ± 0.64 ^a^	2.50 ± 0.42 ^a^
9-Octadecynoate acid	0.33 ± 0.04 ^a^	0.30 ± 0.19 ^a^	-	-
Oleic acid	0.41 ± 0.04 ^b^	0.74 ± 0.05 ^a^	-	-
10-Hydroxy-octadecanoic acid	4.70 ± 0.41 ^a^	2.91 ± 0.09 ^b^	0.34 ± 0.07 ^c^	0.20 ± 0.03 ^c^
17-Methoxy-octadecanoic acid	0.28 ± 0.03	-	-	-
(Z)-11-Eicosenoic acid	-	0.46 ± 0.01 ^b^	0.86 ± 0.07 ^a^	0.30 ± 0.02 ^b^
5,9-Octadecadienoate acid	-	-	0.44 ± 0.08	-
6,10-Octadecadienoate acid	-	-	0.51 ± 0.19	-
Total	57.02 ± 1.01 ^d^	77.91 ± 2.84 ^c^	99.57 ± 2.42 ^b^	107.98 ± 1.6 ^a^

Values are given as the mean ± standard deviation (*n* = 3), and the different letters within each row were significantly different (*p* < 0.05). -: Not detected. Statistically significant differences (*p* < 0.05) were determined by one-way ANOVA with Duncan’s test and were indicated with different letters.

## Data Availability

Not applicable.

## Supplementary Materials

The following supporting information can be downloaded at: https://www.mdpi.com/article/10.3390/molecules27092664/s1. Figure S1: Cell viabilities of *I. terricola* WJL-G4 under different pH (A) and citric acid concentrations (B). Figure S2: PI and DCFH-DA staining observation results.
