# Supplementary material for: Responses of Issatchenkia terricola WJL-G4 upon Citric Acid Stress
Source: Molecules. 2022 Apr 21;27(9):2664. doi: 10.3390/molecules27092664 (PMC9102369; doi:10.3390/molecules27092664)
Supplement: Supplementary file 1 [file molecules-27-02664-s001.zip › molecules-1674182-supplementary.pdf]

(**A**)

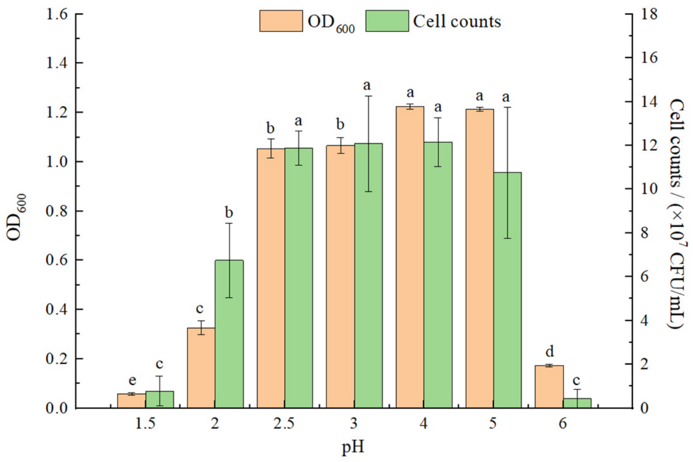

(**B**)

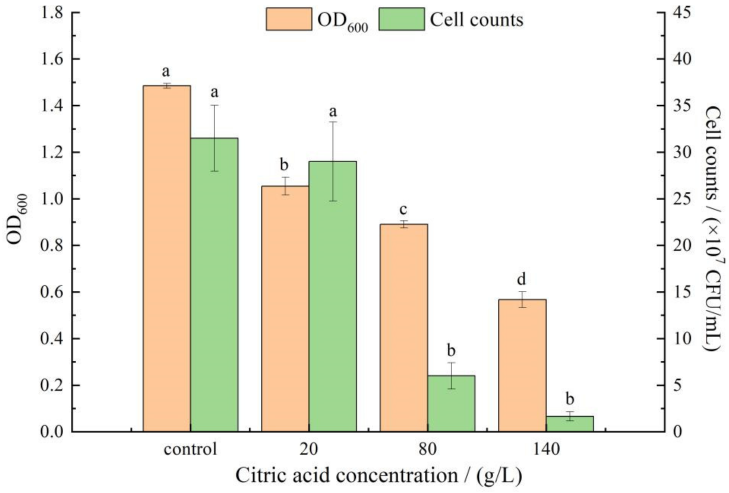

Supplementary Figure S1. Cell viabilities of *I. terricola* WJL-G4 under different pH (**A**) and citric acid concentrations (**B**). Error bars: SD (n = 3). Statistically significant differences ($p < 0.05$) were determined by one-way ANOVA with Duncan's test and were indicated with different letters within each indicator.

(**A**)

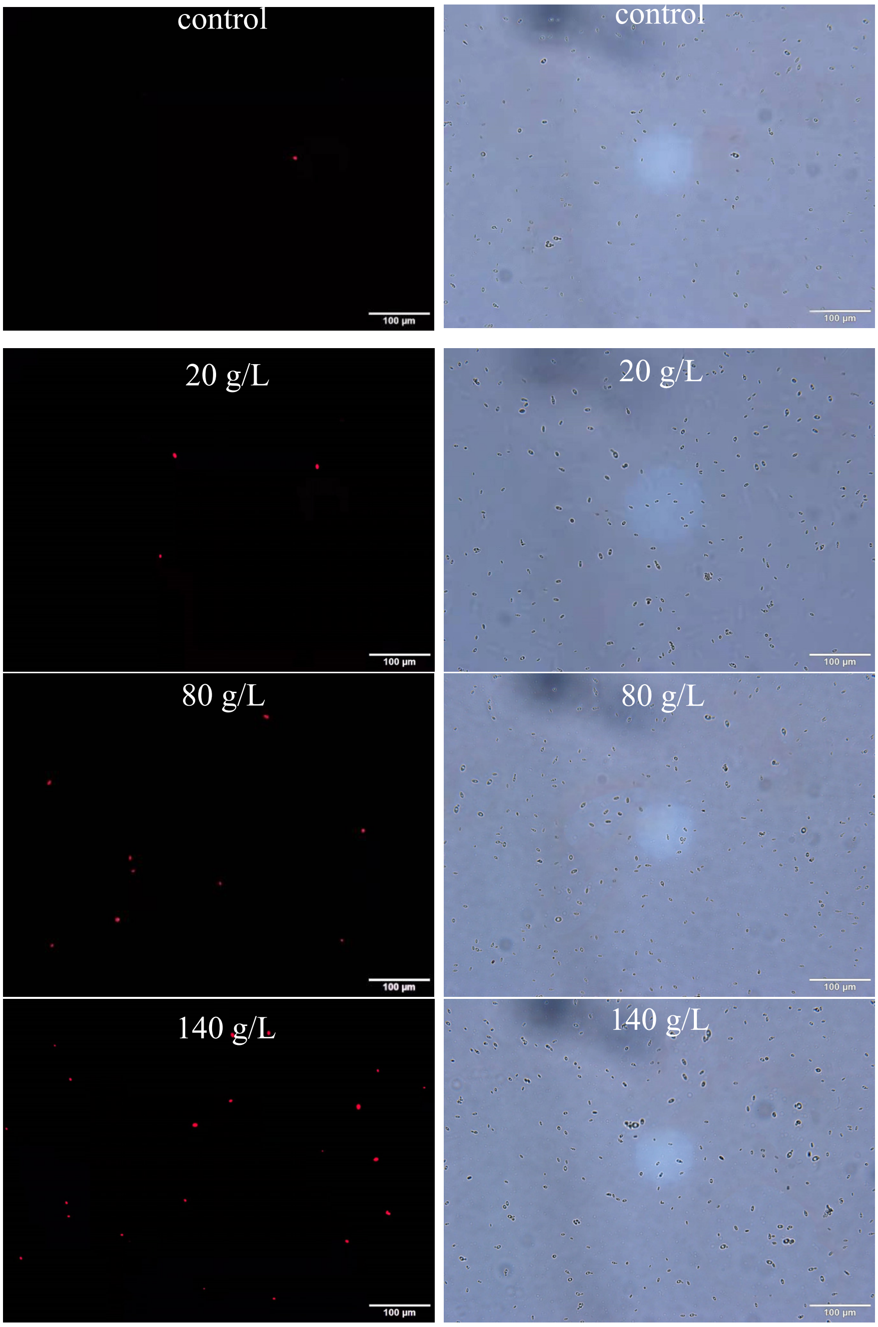

(**B**)

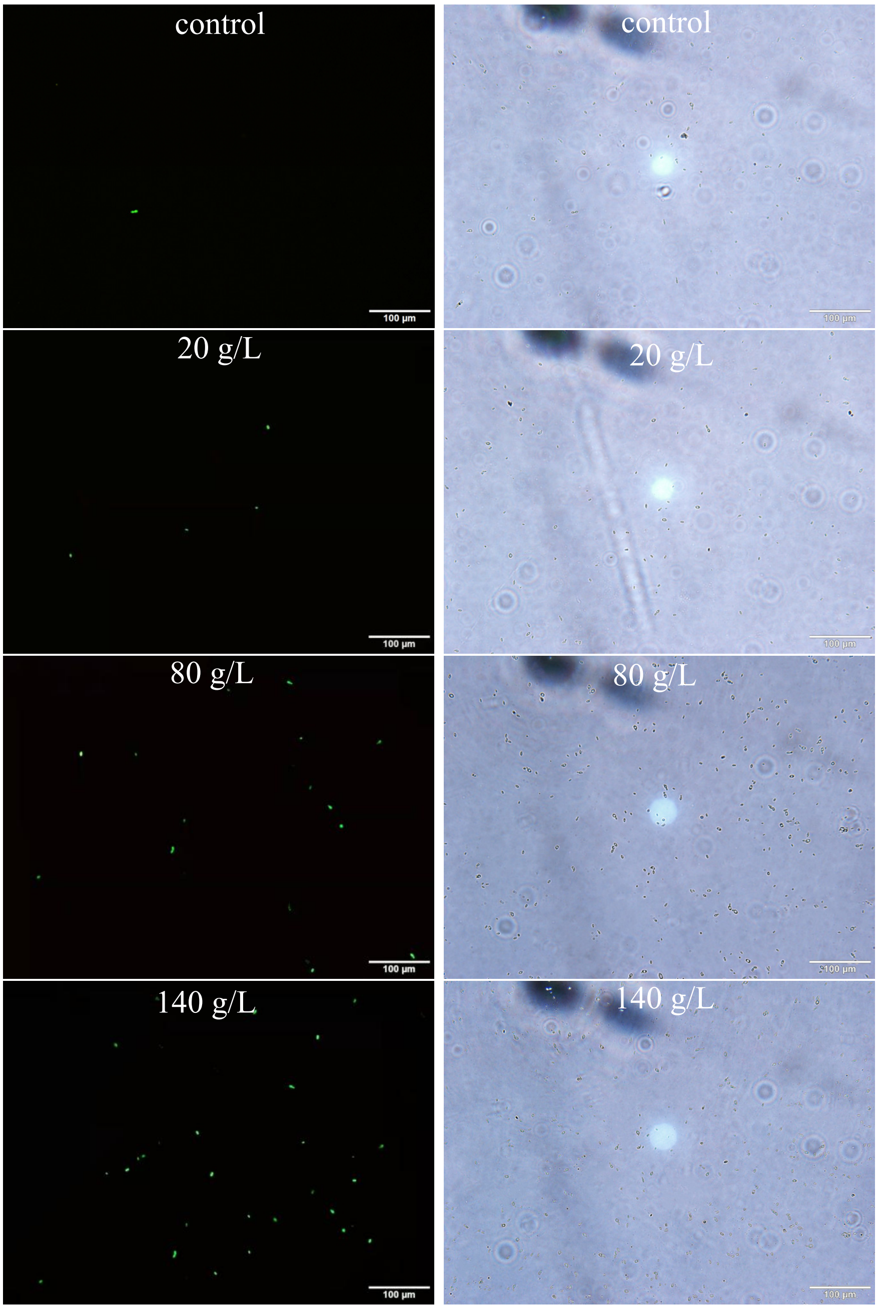

Supplementary Figure S2. PI and DCFH-DA staining observation results. Letters correspond to: (**A**): Fluorescence microscope observation pictures after PI staining. (**B**): Fluorescence microscope observation pictures after DCFH-DA staining.
